# Torrenticolid water mites from Korea and the Russian Far East

**DOI:** 10.3897/zookeys.299.5272

**Published:** 2013-05-14

**Authors:** Vladimir Pešić, Ksenia A. Semenchenko, Wonchoel Lee

**Affiliations:** 1 Department of Biology, University of Montenegro, Cetinjski put b.b., 81000 Podgorica, Montenegro; 2 Institute of Biology and Soil Science, Far Eastern Branch of Russian Academy of Sciences, Vladivostok, 690022 Russia; 3 Hanyang University, Department of Life Sciences, Seoul 133-791, Korea

**Keywords:** Acari, Hydrachnidia, new species, new records, running waters

## Abstract

New records of water mites of the family Torrenticolidae Piersig, 1902 (Acari: Hydrachnidia) from streams in South Korea and the Russian Far East are presented. Detailed descriptions or redescrptions are provided for eight species of the genera *Torrenticola* Piersig, 1896 and *Monatractides* K. Viets 1926. Two species are described as new to science: *Torrenticola kimichungi* **sp. n.** and *Monatractides abei* **sp. n.** Five species are reported as first records from Korea: *Torrenticola brevirostris* (Halbert, 1911); *Torrenticola dentifera* Wiles, 1991; *Torrenticola recentis* Tuzovskij, 2003; *Torrenticola ussuriensis* (Sokolow, 1934); and *Torrenticola turkestanica* (Sokolow, 1926). *Torrenticola nipponica* (Enami, 1940) is reported for the first time from Russia.

## Introduction

Water mites of the family Torrenticolidae are presently known from all continents except Antarctica, with more than 400 species described so far (Zhang et al. 2011, Pešić and Smit 2012a, b, Pešić et al. 2012). However, as the family has its maximum diversity in the still strongly understudied tropical areas, the species number is possibly higher by one order of magnitude (Pešić et al. 2012b). In general, torrenticolid mites are heavily sclerotized, dorso-ventrally flattened, crawling species which colonize running waters with well oxygenated sand and gravel substrates where proto- and tritonymphs can survive the quiescent phase of their life cycle (Di Sabatino et al. 2003).

At present, only one species of the genus *Torrenticola* Piersig, 1896, i.e. *Torrenticola nipponica* (Enami, 1940) is known from South Korea (Chung and Kim 1995), while five species, i.e., *Torrenticola abbreviata* (Sokolow, 1934), *Torrenticola amplexa* (Koenike, 1908), *Torrenticola elliptica* Maglio, 1909, *Torrenticola recentis* Tuzovskij, 2003 and *Torrenticola ussuriensis* (Sokolow, 1934), are known from the Russian Far East (Sokolow 1934, Semenchenko 2010). During a recent survey many specimens of the family Torrenticolidaewere collected throughout South Korea and identification of this material was entrusted to the senior author. This research is part of the project aimed at uncovering Korean invertebrate diversity, and led by the National Institute of Biological Resources (NIBR). The identification of the material from the Russian Far East was made by second author. Eight species of the genera *Torrenticola* Piersig, 1896 and *Monatractides* K. Viets, 1926 are identified, two of them are new to science. Descriptions and redescriptions of these species are given in this paper.

## Material and methods

Water mites were collected by hand netting, sorted on the spot from the living material, preserved in Koenike’s fluid and dissected as described elsewhere (e.g. Gerecke et al. 2007). One sample from the Tigrovaya River (Russian Far East) was obtained via a hand-pump (similar to the Bou-Rouch method) from subterranean waters. A metal tube was hammered into river sediments to a depth of about 30 cm. Pumped samples were filtered through the hand net and fixed in 70 % ethanol for further examination in the laboratory under a stereo microscope. Holotypes and some paratypes are deposited in the National Institute of Biological Resources, Korea (NIBR); other paratypes (material from the Russian Far East) in the research collections of the Institute of Biology and Soil Science, Vladivostok, Russia (IBSS).

In the section ‘Material examined’ collecting site abbreviations derive from the geographical database Pešić. The composition of the material is given as: males/females/deutonymphs. All measurements are given in μm. The following abbreviations are used: asl = above sea level, Cx-I = first coxae, Cxgl-4 = coxoglandularia of fourth coxae (= E4 in Wiles 1997), dL = dorsal length, L = length, I-Leg-6 = Leg 1, sixth segment (tarsus), mL = medial length, Mt = mountain, n = number of specimens examined, NP = National Park, P-1 = palp, first segment, vL = ventral length, W = width.

## Systematics

### Family Torrenticolidae Piersig, 1902 Genus *Torrenticola* Persig, 1896 Subgenus *Torrenticola* Persig, 1896

#### Torrenticola brevirostris

(Halbert, 1911)

http://species-id.net/wiki/Torrenticola_brevirostris

Figs 1 , 7A

Atractides brevirostris Halbert, 1911: 16.

##### Material examined.

SOUTH KOREA: CR9 Ne myeon Mt, Naebyeansan NP, stream near Naebyeansan Info Center, 35°38'25.623"N, 126°34'53.1438"E, 10.x.2012, Pešić & Choi 1/0/0 (mounted, NIBRIV0000268844).

##### Morphology.

**Figure 1.**
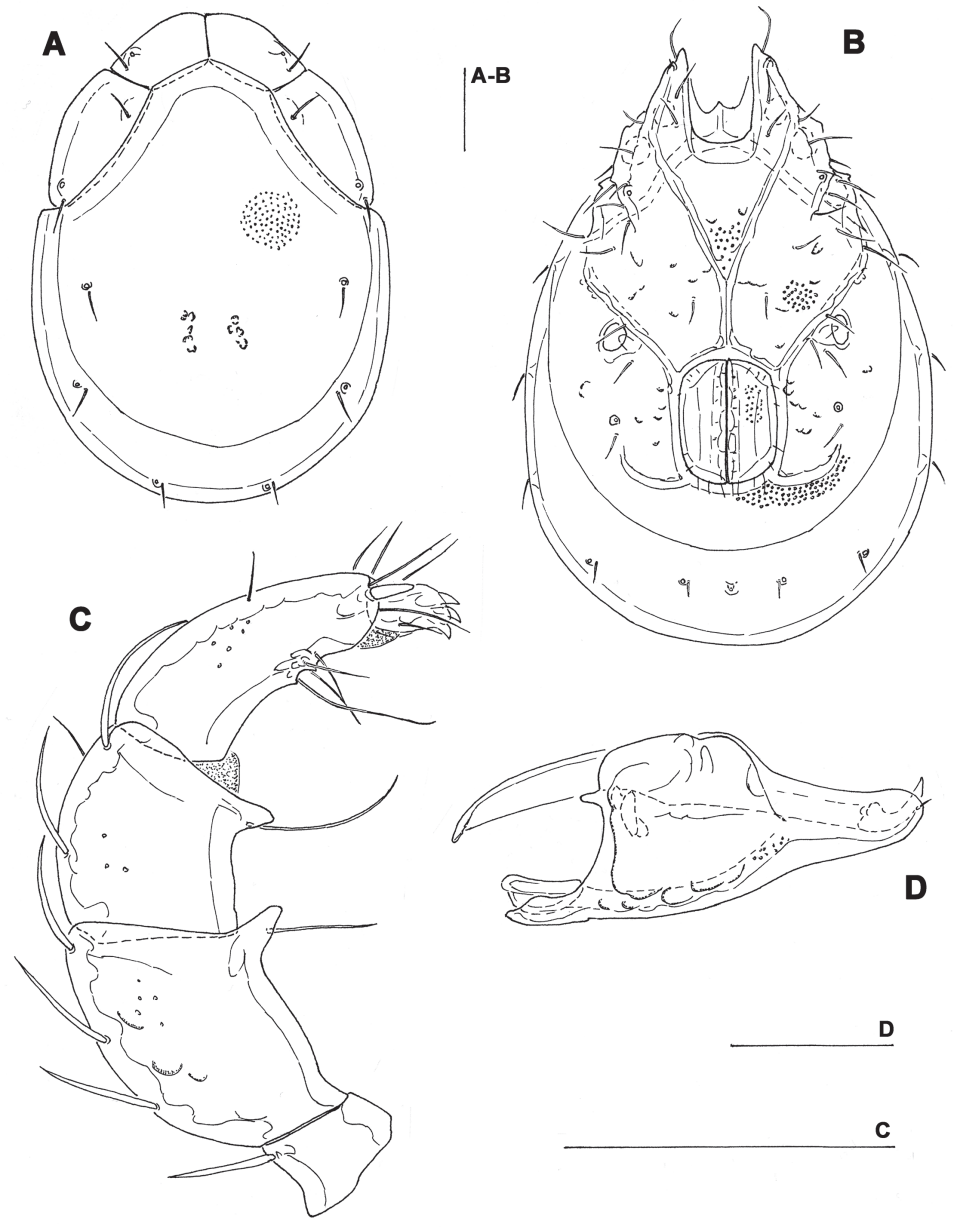
*Torrenticola brevirostris* (Halbert, 1911), male, stream in Naebyeansan NP, Korea: **A** dorsal shield **B** ventral shield **C** palp, medial view **D** gnathosoma. Scale bars = 100 μm.

Male. *General features*. Idiosoma roundish; Cxgl-4 subapical; posterior suture line of Cx-IV starting at right angle from genital field; excretory pore and Vgl-2 away from the line of primary sclerotization (Fig. 1B); ejaculatory complex conventional in shape (with well developed anterior keel and proximal arms); gnathosomal rostrum shortened, ventrally not evidently set off from gathosomal base (Fig. 1D); P-2 shorter than P-4, P-2 ventral margin slightly convex; P-4 stout, with well developed ventral tubercles (Fig. 1C).

*Measurements*. Idiosoma (ventral view: Fig. 1B) L 731, W 500; dorsal shield (Fig. 1A, 7A) L 598, W 441, L/W ratio 1.36; dorsal plate L 544; shoulder plate L 175-177, W 78-83, L/W ratio 2.1-2.3; frontal plate L 130-131, W 66-67, L/W ratio 1.9-2.0; shoulder/frontal plate L ratio 1.34-1.36. Gnathosomal bay L 119, Cx-I total L 284, Cx-I mL 164, Cx-II+III mL 93; ratio Cx-I L/Cx-II+III mL 3.05; Cx-I mL/Cx-II+III mL 1.8. Genital field L/W 152/119, ratio 1.28; ejaculatory complex L 222; distance genital field-excretory pore 127, genital field-caudal idiosoma margin 200. Gnathosoma vL 266; chelicera total L 292; palp total L 296, dL: P-1, 26; P-2, 87; P-3, 66; P-4, 89; P-5, 28; P-2/P-4 ratio 0.98.

##### Remarks.

The single male specimen examined from a stream in Naebyeansan National Park fits well the original description of *Torrenticola brevirostris*. The differences are found in a minor idiosoma and gnathosoma dimensions and a more shallow gnathosoma with a relatively less shortened rostrum compared with the populations from the Western Palaearctic (see: Cicolani and Di Sabatino 1990, Pešić et al. 2006, Di Sabatino et al. 2010). In the shape of gnathosoma the specimen from Korea matches the description of *Torrenticola brevirostris* from Gifu Prefecture in Japan (Imamura 1953). This may suggest that there is some degree of genetic isolation between the populations from the Far East and populations from the Western Palaearctic. However, understanding of these populations is not possible without additional material and probably will require the application of molecular techniques.

##### Habitat.

A permanent sandy/bouldary stream, shaded by riparian vegetation (Fig. 14B).

##### Distribution.

Palaearctic. New for the fauna of Korea.

#### Torrenticola dentifera

Wiles, 1991

http://species-id.net/wiki/Torrenticola_dentifera

Figs 2 , 7B

Torrenticola dentifera Wiles, 1991: 43.

##### Material examined.

SOUTH KOREA: CR9 Ne myeon Mt, Naebyeansan NP, stream near Naebyeansan Info Center, 35°38'25.623"N, 126°34'53.1438"E, 10.x.2012, Pešić & Choi 1/0/0 (mounted, NIBRIV0000268845).

##### Morphology.

**Figure 2.**
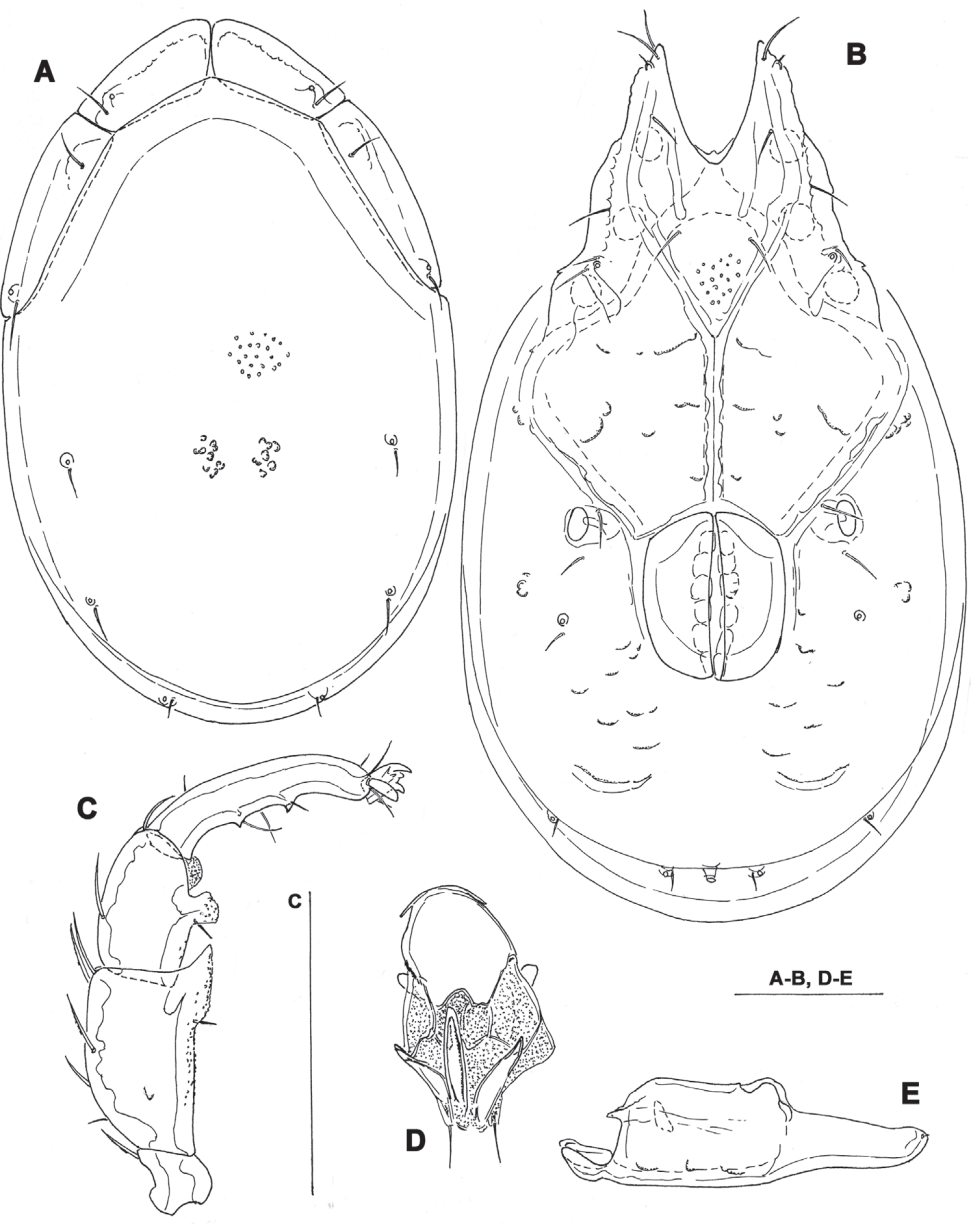
*Torrenticola dentifera* Wiles, 1991, male, stream in Naebyeansan NP, Korea: **A** dorsal shield **B** ventral shield **C** palp, medial view **D** ejaculatory complex **E** gnathosoma. Scale bars = 100 μm.

Male. *General features*. Idiosoma elongated; frontal platelets anteriorly bulging (Figs 2A, 7B); Cxgl-4 subapical; medial suture line of Cx-II+III relatively long; posterior suture line of Cx-IV weakly curved; excretory pore and Vgl-2 on the line of primary sclerotization near posterior idiosoma margin; ejaculatory complex conventional in shape (Fig. 2D); gnathosoma ventral margin only slightly curved, rostrum well developed (Fig. 2E); P-2 shorter than P-4, ventral margin with a fine denticulation also in proximal half of the segment, distally with a laterally compressed, anteriorly directed hyaline extension; P-3 with a subrectangular, apically serrated ventrodistal projection with a fine denticles; P-4 with long and broadly rounded distal seta (Fig. 2C), ventral tubercles well developed and separated.

*Measurements*. Idiosoma (ventral view: Fig. 2B) L 591, W 341; dorsal shield (Figs 2A, 7B) L 472, W 305, L/W ratio 1.55; dorsal plate L 434; shoulder plate L 141-144, W 37-40, L/W ratio 3.6-3.8; frontal plate L 101-108, W 47-48, L/W ratio 2.2-2.3; shoulder/frontal plate L ratio 1.3-1.4. Gnathosomal bay L 81, Cx-I total L 200, Cx-I mL 117, Cx-II+III mL 113; ratio Cx-I L/Cx-II+III mL 1.8; Cx-I mL/Cx-II+III mL 1.04. Genital field L/W 113/95, ratio 1.19; ejaculatory complex L 166; distance genital field-excretory pore 133, genital field-caudal idiosoma margin 161. Gnathosoma vL 247; palp total L 226, dL: P-1, 22; P-2, 62; P-3, 48; P-4, 77; P-5, 17; P-2/P-4 ratio 0.8.

##### Remarks.

The single male specimen examined fits the original description of *Torrenticola dentifera*,which was based on two male specimens from Selangor, Peninsular Malaysia (Wiles 1991). The only differences are found in larger dimensions of idiosoma and palps of the South Korea specimen.

##### Habitat.

A permanent sandy/bouldary stream, shaded by riparian vegetation (Fig. 14B).

##### Distribution.

Malaysia (Wiles 1991, 1997). New for the fauna of Korea.

#### Torrenticola kimichungi sp. n.

urn:lsid:zoobank.org:act:B92491ED-F051-4C8B-9381-BFC4668BA2CE

http://species-id.net/wiki/Torrenticola_kimichungi

Figs 3 , 4 , 7C–F

##### Type series.

Holotype male (NIBRIV0000268846), dissected and slide mounted, SOUTH KOREA: CR4 Seoraksan NP, stream near Temple, 38°10.399'N, 128°29.050'E, 196 m asl., 8.x.2012 Pešić & Karanović. Paratype: same data as holotype, one male (NIBRIV0000268847); RUSSİA, Primory Territory, Partizansky District, Partizanskay River basin, Tigrovaya River, 43°11.401'N, 133°12.660'E; depth 30 cm below the sediment surface; substrate: cobbles, pebbles, sand; 12.vi.2010, Semenchenko & Sidorov, three males (490-492-kas–IBSS), two females (493-494-kas–IBSS), dissected and slide mounted.

##### Diagnosis.

Idiosoma elongated (dorsal shield L/W ratio 1.5-1.6); medial suture line of Cx-II+III in male short (L 74-85 μm); suture line of Cx-IV extended posterior to the genital field; excretory pore posterior to the line of primary sclerotization, Vgl-2 posterior to excretory pore; male genital field with maximum width at the anterior margin; gnathosoma deep with a short rostrum; P-4 with ventral setae on flat hump.

##### Description.

**Figure 3.**
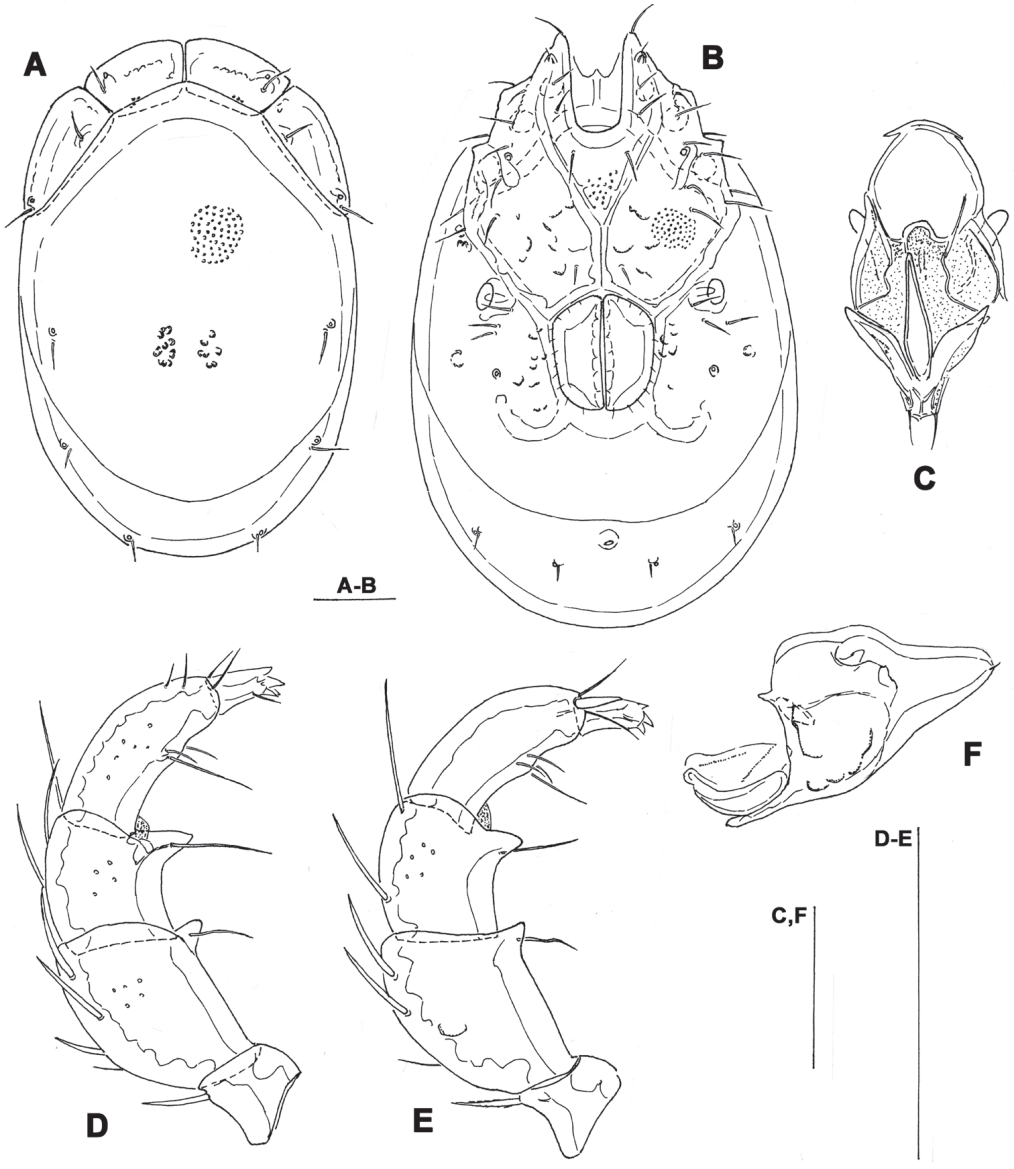
*Torrenticola kimichungi* sp. n., male holotype: **A** dorsal shield **B** ventral shield **C** ejaculatory complex **D** palp, lateral view **E** palp, medial view **F** gnathosoma. Scale bars = 100 μm.

**Figure 4.**
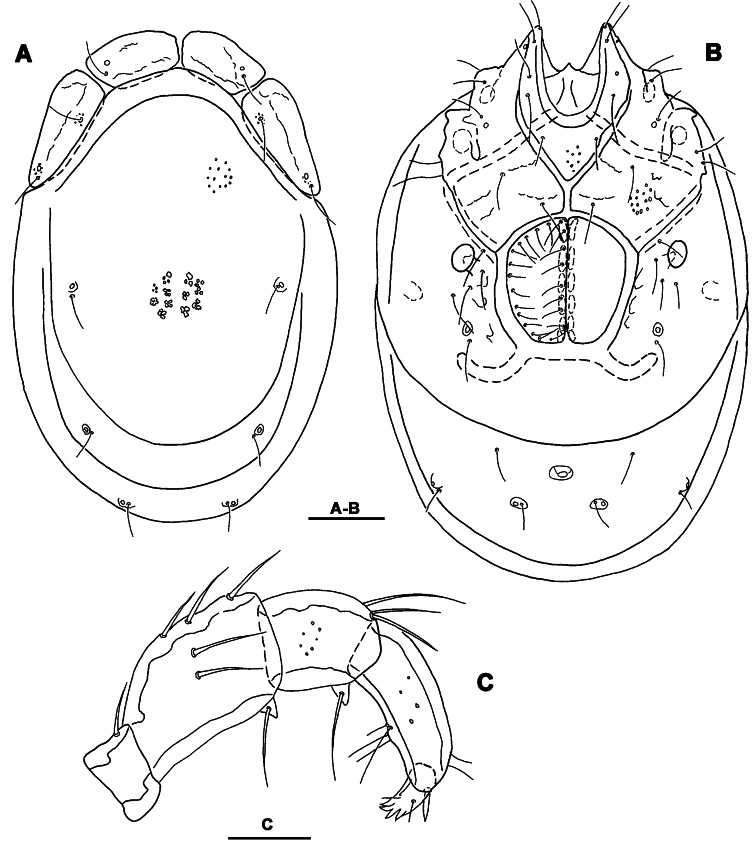
*Torrenticola kimichungi* sp. n., paratype female, Tigrovaya River, Russia: **A** dorsal shield **B** ventral shield **C** palp, lateral view. Scale bars = 100 μm (**A–B**), 25 μm (**C**).

*General features*. Idiosoma elongated; Cxgl-4 subapical; suture line of Cx-IV evident and curved, starting posterior from genital field, laterally curved anteriorly (Figs 3B, 4B); excretory pore posterior to the line of primary sclerotization, Vgl-2 posterior to excretory pore; gnathosoma deep with a short rostrum not evidently set off from gnathosomal base (Fig. 3F); P-2 ventrally slightly convex, ventrodistal projection cone-shaped, pointed towards distal, P-3 with ventrodistal projection slightly larger than projection of P-2, P-4 slightly curved, ventral setae (one long and three short) on flat hump (Figs 3D–E, 4C). Male: Medial suture line of Cx-II+III short; genital field with maximum width at the anterior margin; ejaculatory complex normal in shape (Fig. 3C); P-2 and P-4 almost equal in length.

*Measurements*. Male (holotype, in parentheses measurements of paratype from South Korea, in square parentheses specimens from Russia, n = 2): Idiosoma (ventral view: Fig. 3B) L 723 (741) [731-748], W 477 (513) [488-578]; dorsal shield (Figs 3A, 7C–E) L 628 (650) [607-663], W 414 (444) [409-425], L/W ratio 1.52 (1.46) [1.47-1.56]; dorsal plate 575 (596) [554-595]; shoulder plate L 166-169 (163-166) [161-167], W 72-75 (73) [72-79], L/W ratio 2.2-2.3 (2.2-2.3) [2.11-2.24]; frontal plate L 131-134 (127-131) [125-126], W 72 (69-73) [62-72], L/W ratio 1.8-1.9 (1.7-1.9) [1.75- 2.0]; shoulder/frontal plate L ratio 1.24-1.29 (1.27-1.28) [1.28-1.33]. Gnathosomal bay L 122 (125) [112-119], Cx-I total L 231 (244) [231-237], Cx-I mL 109 (119) [118-119], Cx-II+III mL 75 (85) [74-79]; ratio Cx-I L/Cx-II+III mL 3.1 (2.9) [2.91-3.2]; Cx-I mL/Cx-II+III mL 1.45 (1.4) [1.5-1.58]. Genital field L/W 138 (148) [145-152]/122 (125) [112-114], L/W ratio 1.12 (1.18) [1.26-1.36]; ejaculatory complex L 183 (184) [180-200]; distance genital field-excretory pore 166 (163) [152-172], genital field-caudal idiosoma margin 266 (263) [251-257]. Gnathosoma vL 191 (198) [194-211]; chelicera total L 234 (231) [231-232]; palp total L 215 (210) [210-216], dL: P-1, 25 (21) [24-27]; P-2, 62 (61) [59-62]; P-3, 43 (42) [43-46]; P-4, 60 (63) [62-63]; P-5, 25 (23) [19-21]; P-2/P-4 ratio 1.03 (0.97) [0.94-1.0].

Female (from Russia, n = 2). Idiosoma (ventral view: Fig. 4B) L 800-816, W 544-580; dorsal shield (Figs 4A, 7F) L 714-731, W 476-493, L/W ratio 1.45-1.54; dorsal plate L 650-663; shoulder plate L 174-185, W 72-74, L/W ratio 2.34-2.57; frontal plate L 132-133, W 71-72, L/W ratio 1.83-1.86; shoulder/frontal plate L ratio 1.32-1.4. Gnathosomal bay L 119-132, Cx-I total L 231-244, Cx-I mL 112-113, Cx-II+III mL 39-46; ratio Cx-I L/Cx-II+III mL 5.02-6.25; Cx-I mL/Cx-II+III mL 2.43-2.9. Genital field L/W 174-178/162-165, L/W ratio 1.05-1.09; distance genital field-excretory pore 160-198, genital field-caudal idiosoma margin 310-330. Gnathosoma vL 218-264; chelicera total L 244; palp total L 220-228, dL: P-1, 24-27; P-2, 66-70; P-3, 46-48; P-4, 64-65; P-5, 18-20; P-2/P-4 ratio 1.01-1.08.

##### Etymology.

The species is named after Drs Il-Hoi Kim and Kyung-Sook Chung in appreciation of their studies of the Korean water mites.

##### Remarks.

The new species belongs to the group of species characterized by having well-developed finger or peg-like ventrodistal tubercles on P-2 and P-3, the deep gnathosoma with a short rostrum, not evidently set off from gnathosomal base and a relatively short medial suture line of Cx-II+III in male. This group includes the following Asian species of *Torrenticola*: *Torrenticola brevirostris* (Halbert, 1911) (Palaearctic), *Torrenticola nanshihensis* Pešić et al., 2011 (Taiwan), *Torrenticola projectura* Pešić et al., 2012 (Taiwan), *Torrenticola retractipora* Lundblad, 1969 (Burma), *Torrenticola siamis* Pešić & Smit, 2009 (Thailand), and *Torrenticola subterranea* Imamura, 1957 (Japan). Males of *Torrenticola brevirostris* and *Torrenticola nanshihensis* differ in a prominent suture line of Cx-IV starting at a right angle from the genital field, excretory pore on the same level as Vgl-2 and more away from the line of primary sclerotization and P-4 stockier with well developed ventral tubercles (see: Di Sabatino et al. 2010 for *Torrenticola brevirostris*, and Pešić et al. 2012 for *Torrenticola nanshihensis*). *Torrenticola projectura* clearly separates in having P-3 with a long tapering ventral protrusion which curves distally (Pešić et al. 2012a). Males of *Torrenticola retractipora* can easily be distinguished by larger dimensions of the idiosoma, a differently shaped ejaculatory complex with large proximal chamber (see: Lundblad 1969), and a moderately long median suture line of Cx-II+III (101 μm, data taken from Wiles 1997). *Torrenticola subterranea*, a weakly defined species known from subterranean habitats in Japan (Imamura 1957, 1959) is similar in Cx-IV extended posterior to the genital field, but differs in narrower frontal platelets, excretory pore and Vgl-2 lying on the margin of primary sclerotization, and a less developed distoventral projections on P-2 and P-3 (see: Imamura 1959). *Torrenticola siamis* closely resembles *Torrenticola kimichungi* sp. n. in the general shape of idiosoma and palp, but males are distinguishable in having Cx-IV not extended posterior to the genital field and the genital field more elongated (L/W ratio 1.4, data taken from Pešić and Smit 2009) and rectangular in shape.

##### Habitat.

A permanent sandy/bouldery stream with considerably exposure to sunlight (Fig. 14C); the specimens from Russia were collected from interstitial waters.

##### Distribution.

South Korea, Far East of Russia (present study).

#### Torrenticola nipponica

(Enami, 1940)

http://species-id.net/wiki/Torrenticola_nipponica

Figs 5 , 6 , 7G–I

Atractides nipponicus Enami, 1940: 221.

##### Material examined.

SOUTH KOREA: CR1 Seoul, Dobong stream, 37°41.262'N, 127°01.706'E, 19 m asl., 7.x.2012, Pešić & Choi 2/0/0 (mounted, NIBRIV0000268848). RUSSIA: Primory Territory, Partizansky District, Partizanskay River basin, Tigrovaya River, 43°11.401'N, 133°12.660'E; depth 30 cm below the sediment surface; substrate: cobbles, pebbles, sand; 12.vi.2010, Semenchenko & Sidorov 2/2/0 (mounted, IBSS).

##### Morphology.

**Figure 5.**
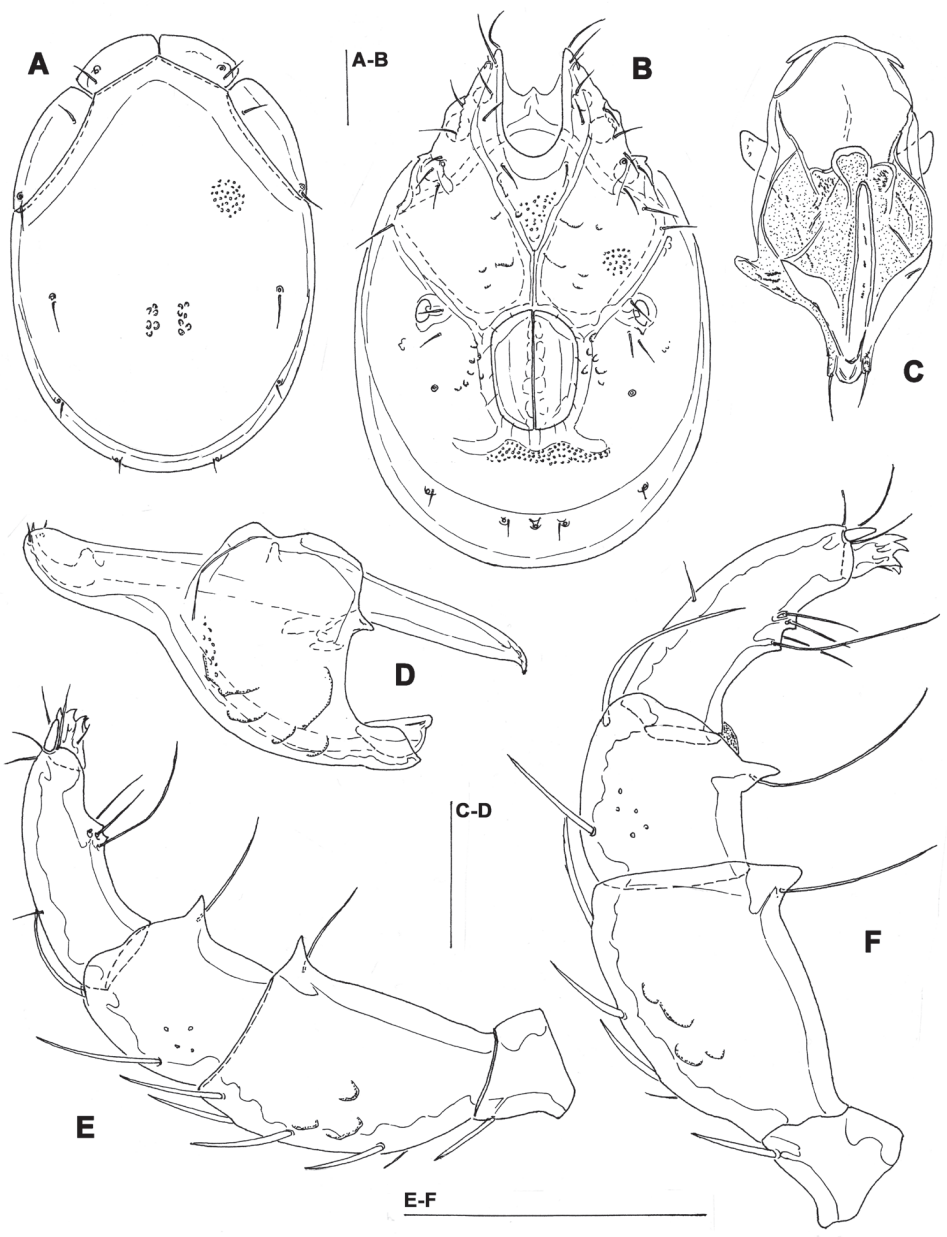
*Torrenticola nipponica* (Enami, 1940), male, Dobong stream, Korea: **A** dorsal shield **B** ventral shield **C** ejaculatory complex **D** gnathosoma **E–F** palp, medial view (E-smaller specimen, F-larger specimen). Scale bars = 100 μm.

**Figure 6.**
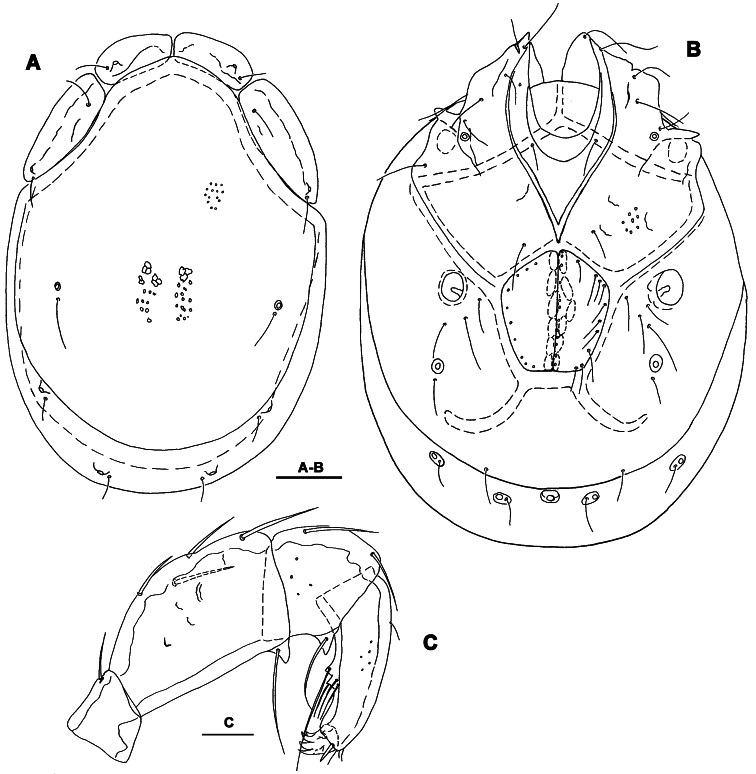
*Torrenticola nipponica* (Enami, 1940), female, Tigrovaya River, Russia: **A** dorsal shield **B** ventral shield **C** palp, lateral view. Scale bars = 100 μm (**A–B**), 25 μm (**C**).

**Figure 7.**
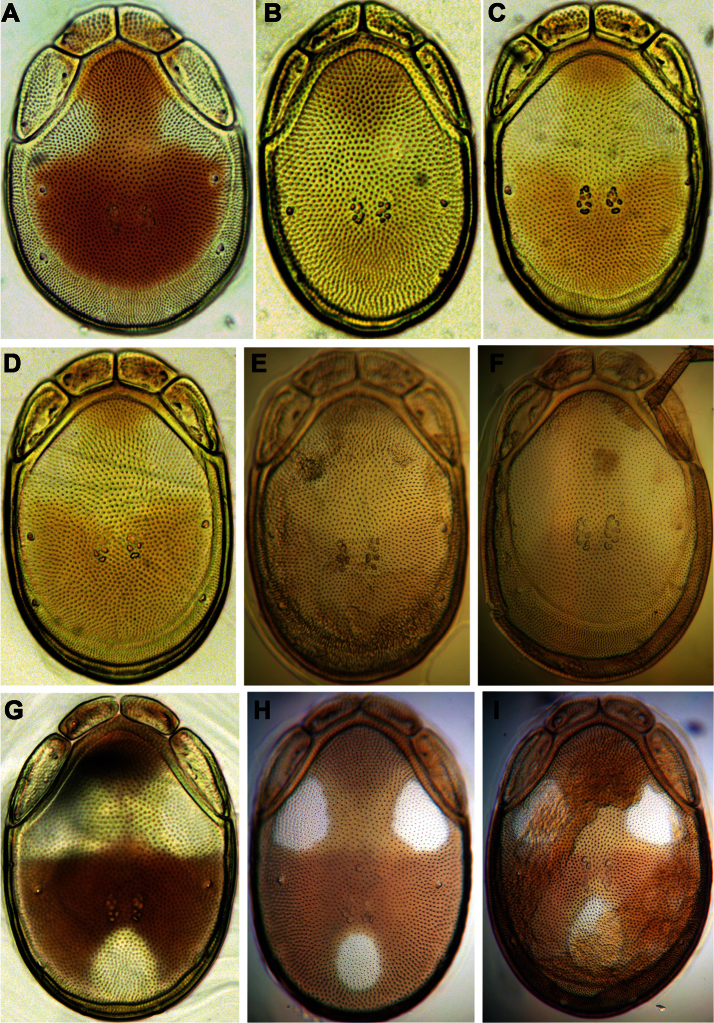
Photographs of dorsal shield: **A** *Torrenticola brevirostris* (Halbert, 1911), female, stream in Naebyeansan NP, Korea **B** *Torrenticola dentifera* Wiles, 1991, male, stream in Naebyeansan NP, Korea: **C–F** *Torrenticola kimichungi* sp. n. (**C–D** specimens from stream in Seoraksan NP, Korea, **E–F** specimens from Tigrovaya River, Russia): **C** maleholotype **D–E** maleparatypes **F** femaleparatype **G–I** *Torrenticola nipponica* (Enami, 1940) (**G** specimen from Dobong stream, Korea **H–I** specimens from Tigrovaya River, Russia): **G–H** male **I** female. Photos. V. Pešić (Figs **A–D, G**), K. Semenchenko (Figs **E–F, H–I**).

*General features*. Idiosoma roundish; dorsal shield with colour pattern as illustrated in Figs 7G-I; Cxgl-4 subapical; excretory pore and Vgl-2 slightly away from the line of primary sclerotization; gnathosoma deep, rostrum shorter than depth of gnathosomal base (Fig. 5D); palp robust and compact, P-2 longer than P-4, P-2 and P-3 ventrodistal projection pointed towards distal, P-4 with well developed ventral tubercles bearing one long and three short setae (Figs 5E-F, 6C). Male: Medial suture line of Cx-II+III short; suture line of Cx-IV medially starting from posterior margin of genital field (Fig. 2B); ejaculatory complex normal in shape (Fig. 5C). Female: Suture of Cx-IV curved (Fig. 2C); genital field pentagonal in shape.

*Measurements*. Male (from South Korea, n = 2; in parentheses specimens from Russia, n = 2) Idiosoma (ventral view: Fig. 5B) L 694-819 (740-755), W 478-534 (508-544); dorsal shield (Figs 5A, 7G-H) L 584-675 (636-670), W 410-469 (422-476), L/W ratio 1.42-1.44 (1.4-1.5); dorsal plate L 556-643 (594-614); shoulder plate L 178-206 (178-188), W 56-61 (59-60), L/W ratio 3.0-3.4 (2.96-3.18); frontal plate L 113-125 (121-125), W 45-50 (46-47), L/W ratio 2.4-2.5 (2.56- 2.72); shoulder/frontal plate L ratio 1.56-1.67 (1.46-1.5). Gnathosomal bay L 148-153 (132-141), Cx-I total L 278-294 (284-286), Cx-I mL 129-141 (145-165), Cx-II+III mL 69-91 (79-92); ratio Cx-I L/Cx-II+III mL 3.2-4.0 (3.1-3.6); Cx-I mL/Cx-II+III mL 1.55-1.87 (1.57-2.08). Genital field L/W 156-181/117-133 (168-172/125-132), L/W ratio 1.33-1.36 (1.26-1.38); ejaculatory complex L 231 (224-251); distance genital field-excretory pore 131-178 (138-145), genital field-caudal idiosoma margin 183-231 (172-205). Gnathosoma vL 273-303 (277-297); chelicera total L 319-347 (323-343); palp total L 279-316 (284-291), dL: P-1, 34-37 (33-36); P-2, 92-101 (92-95); P-3, 52-63 (51-59); P-4, 82-93 (82-92); P-5, 19-22 (17-18); P-2/P-4 ratio 1.09-1.13 (1.03-1.12).

Female (from Russia, n = 2). Idiosoma (ventral view: Fig. 6B) L 860-867, W 629-635; dorsal shield (Figs 6A, 7I) L 723-782, W 502-544, L/W ratio 1.44; dorsal plate L 680-731; shoulder plate L 211-214, W 66-73, L/W ratio 2.92-3.2; frontal plate L 128-139, W 59-64, L/W ratio 2.16- 2.17; shoulder/frontal plate L ratio 1.54-1.65. Gnathosomal bay L 168-178, Cx-I total L 303-310, Cx-I mL 132-145, Cx-II+III mL 30-40; ratio Cx-I L/Cx-II+III mL 7.75-10.1; Cx-I mL/Cx-II+III mL 3.63-4.4. Genital field L/W 185-195/172-178, L/W ratio 1.04-1.13; distance genital field-excretory pore 208-210, genital field-caudal idiosoma margin 297-310. Gnathosoma vL 330-336; chelicera total L 420; palp total L 341-345, dL: P-1, 41-43; P-2, 115-116; P-3, 65-67; P-4, 100-102; P-5, 18-19; P-2/P-4 ratio 1.13-1.16.

##### Remarks.

The male specimens from South Korea and Russia fit the description of *Torrenticola nipponica* (Enami, 1940) which was based on one male and seven females from River Inôzava, Uzi region in Japan (Enami 1940). However, as the type material was probably lost (not found in the arachnid collection in the National Museum of Nature and Science, Tokyo, Hirotsugu Ono pers. comm.) additional sampling and selection of a neotype from the locus typicus is necessary to guarantee taxonomic stability of *Torrenticola nipponica*. In the original description, Enami (1940) compared *Torrenticola nipponica* with *Torrenticola brevirostris* (Halbert, 1911), a species which differs in the gnathosomal rostrum not distinctly set off from the gnathosomal base, P-2 shorter than P-4, the suture line of Cx-IV starting at right angle from the genital field, the excretory pore and Vgl-2 more distanced from the line of primary sclerotization and the genital field in male is less elongated.

##### Habitat.

A permanent shaded sandy/bouldary stream at low elevations (Fig. 14A); the specimens from Russia were collected from interstitial waters.

##### Distribution.

Japan (Uzi region- Enami, 1940), South Korea (Chindo Island – Chung & Kim 1995; present study). New for the fauna of Russia.

#### Torrenticola recentis

Tuzovskij, 2003

http://species-id.net/wiki/Torrenticola_recentis

Figs 8 , 11A–C

Torrenticola recentis Tuzovskij, 2003: 45.4

##### Material examined.

SOUTH KOREA: CR1 Seoul, Dobong stream, 37°41.262'N, 127°01.706'E, 19 m asl., 7.x.2012, Pešić & Choi 6/6/0 (1/1/0 mounted, NIBR IV0000268849); CR2 Seoul, Ui-dong stream 37°39.554'N, 127°00.249'E, 114 m asl., 7.x.2012, Pešić & Choi 3[1 juvenile]/0/1; CR4 Seoraksan NP, stream near Temple, 38°10.399'N, 128°29.050'E, 196 m asl., 8.x.2012 Pešić & Karanović 7/2/5; CR7 Odesean NP, stream, 37°49.642'N, 128°42.170'E, 215 m asl., 9.x.2012, Pešić & Karanović 6/6/0 (2/1/0 mounted, NIBRIV0000268850);; CR11 Mudeung Mt., stream, 35°8'50.2584"N, 126°59'18.942"E, 11.x.2012, Pešić & Choi 2/2/1; CR12 JiriSan NP, stream near waterfall, 35°22'47''N, 127°29'10''E, 11.x.2012, Pešić & Choi 4/12[1 juvenile]/0 (1/0/0 mounted); CR14 Duckyu San NP, stream, 35°53'50"N, 127°46'35"E, 11.x.2012, Pešić & Choi 0/2/0. RUSSIA, Primory Territory, Khasansky District, “Kedrovaya Pad National Nature Biosphere Reserve”, Sea of Japan basin, Kedrovaya River, 43°06.056'N, 131°33.310'E; depth 12–50 cm; substrate: boulders, cobbles, pebbles; 8.xi.1993, Tiunova 2/2/0 (IBSS).

##### Morphology.

**Figure 8.**
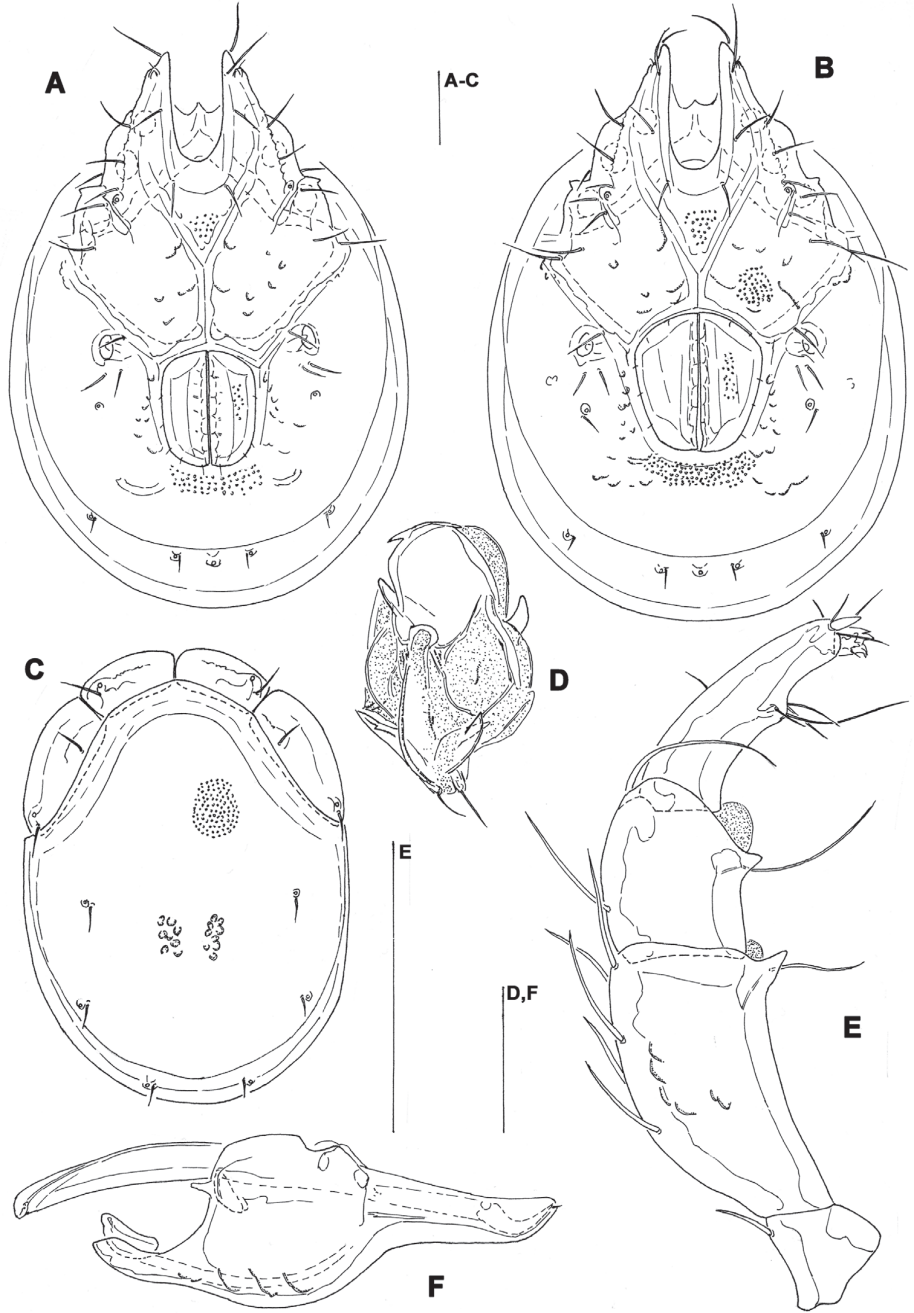
*Torrenticola recentis* Tuzovskij, 2003 (**A, C–F** male **B** female), Dobong stream, Korea: **A–B** ventral shield **C** dorsal shield **D** ejaculatory complex **E** palp, medial view **F** gnathosoma and chelicera. Scale bars = 100 μm.

*General features*. Idiosoma roundish; dorsal shield with colour pattern as illustrated in Figs 11A–C; Cxgl-4 subapical; suture line of Cx-IV hardly evident; excretory pore and Vgl-2 only slightly away from the line of primary sclerotization (Fig. 8A); gnathosoma ventral margin strongly curved (Fig. 8F); P-2 longer than P-4, P-2 ventral margin slightly concave, P-4 with well developed ventral protuberance bearing one long and three short setae (Fig. 8E). Male: Medial suture line of Cx-II+III moderately long; genital field subrectangular in shape, ejaculatory complex with small proximal chamber and robust proximal arms (Fig. 8D).

*Measurements*. Male (from CR1, in parentheses specimen from CR7). Idiosoma (ventral view: Fig. 7A) L 769 (753), W 556 (522); dorsal shield (Figs 7C, 11A-B) L 631 (613), W 450 (446), L/W ratio 1.4 (1.4); dorsal plate L 589 (575); shoulder plate L 195-202 (188-195), W 78 (70-72), L/W ratio 2.5-2.6 (2.6-2.8); frontal plate L 134-138 (136-138), W 58-63 (55-56), L/W ratio 2.1-2.4 (2.4-2.5); shoulder/frontal plate L ratio 1.4-1.5 (1.4). Gnathosomal bay L 152 (159), Cx-I total L 292 (306), Cx-I mL 141 (147), Cx-II+III mL 117 (103); ratio Cx-I L/Cx-II+III mL 2.5 (3.0); Cx-I mL/Cx-II+III mL 1.2 (1.4). Genital field L/W 161 (150) /129 (125), ratio 1.25 (1.2); ejaculatory complex L 189 (193); distance genital field-excretory pore 131 (134), genital field-caudal idiosoma margin 198 (191). Gnathosoma vL 325 (320); chelicera total L 376 (381); palp total L 298 (285), dL: P-1, 34 (34); P-2, 104 (97); P-3, 55 (52); P-4, 91 (88); P-5, 14 (14); P-2/P-4 ratio 1.14 (1.1).

Female (from CR1). Idiosoma (ventral view: Fig. 7B) L 825, W 569; dorsal shield (Fig. 11X) L 672, W 488, L/W ratio 1.38; dorsal plate L 631; shoulder plate L 212-213, W 69, L/W ratio 3.1; frontal plate L 142-144, W 56-59, L/W ratio 2.4-2.5; shoulder/frontal plate L ratio 1.5. Gnathosomal bay L 179, Cx-I total L 320, Cx-I mL 141, Cx-II+III mL 47; ratio Cx-I L/Cx-II+III mL 6.8; Cx-I mL/Cx-II+III mL 3.0. Genital field L/W 191/176, ratio 1.09; egg (n = 1) maximum diameter 222; distance genital field-excretory pore 163, genital field-caudal idiosoma margin 250. Gnathosoma vL 344; chelicera total L 404; palp total L 318-321, dL: P-1, 39; P-2, 110-112; P-3, 56-57; P-4, 97; P-5, 16; P-2/P-4 ratio 1.13-1.16.

##### Remarks.

The specimens from South Korea fit the description of *Torrenticola recentis*,a species described by Tuzovskij (2003) from the Primory Territory in the Russian Far East, and later on reported by Semenchenko (2006) from the River Kedrovaya in the southern part of Primory Territory and from many other southern and northern rivers in the Primory Territory (Semenchenko 2010). The specimens examined from River Kedrovaya agrees well with our specimens due to colour pattern of dorsal shield and length of medial suture line of Cx-II+III, characters not given by Tuzovskij (2003) in the original description of *Torrenticola recentis*.

*Torrenticola elliptica* Maglio, 1909, a species similar in general shape of idiosioma and palps, differs from *Torrenticola recentis* in a more slender idiosoma, a more extended postgenital area in the male and the ejaculatory complex with a large proximal chamber (see: Di Sabatino et al. 2010). Possibly *Torrenticola recentis* was misidentified as *Torrenticola elliptica* in many old records from the Russian Primory Territory (e.g. Sokolow 1934). Likewise, old records from Japan (Enami 1940) might refer to *Torrenticola recentis*.

##### Habitat.

Running waters at low and middle elevations (Figs 14A,D, F).

##### Distribution.

Far East of Russia (Primory Territory – Tuzovskij 2003, Semenchenko 2006, 2010). New for the fauna of Korea.

#### Torrenticola ussuriensis

(Sokolow, 1940)

http://species-id.net/wiki/Torrenticola_ussuriensis

Figs 9 , 411D–F

Atractides ussuriensis Sokolow, 1940: 347.

##### Material examined.

SOUTH KOREA: CR3 River Inje, 38°03.961'N, 128°10.516'E, 225 m asl., 8.x.2012 Pešić & Karanović 0/1/0 (mounted, NIBRIV0000268851).

##### Morphology.

**Figure 9.**
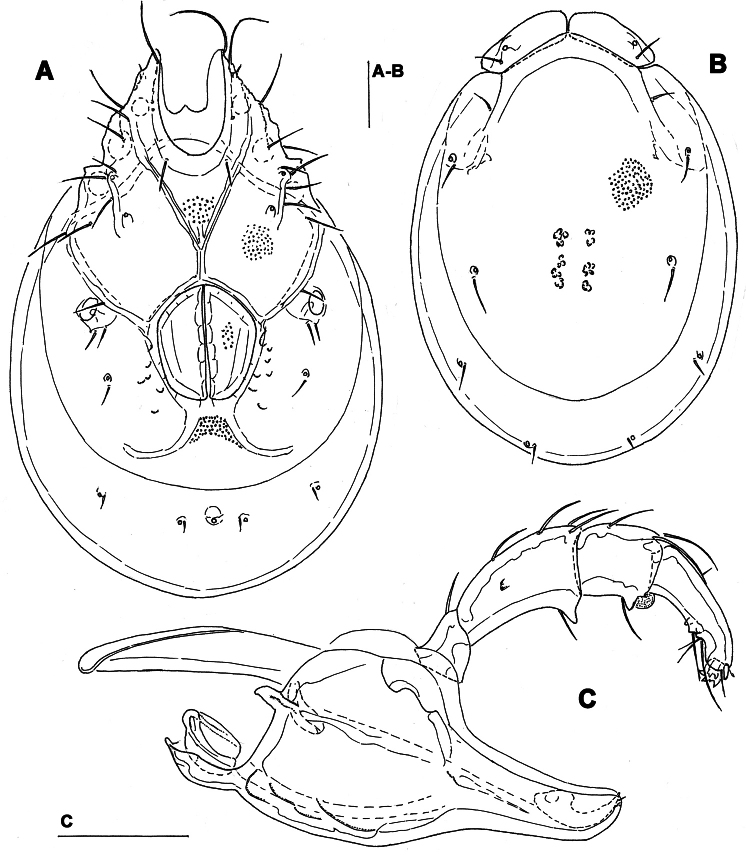
*Torrenticola ussuriensis* (Sokolow, 1940), female, Inje River, Korea: **A** ventral shield **B** dorsal shield **C** gnathosoma and palp, medial view. Scale bars = 100 μm.

Female. *General features*. Shoulder platelets fused to the large dorsal plate (Fig. 9B); Cxgl-4 posterior to Cxgl-2 (Fig. 9A), glandular pore Cxgl-4 distanced from Cxgl-2 by 50-55 µm; excretory pore and Vgl-2 clearly posterior to the line of primary sclerotization; suture of Cx-IV curved; P-2 longer than P-4; P-4 with four well developed ventral tubercles (Fig. 9C).

*Measurements*. Idiosoma (ventral view: Fig. 9A) L 838, W 569; dorsal shield (Fig. 9B, 11D-E) L 694, W 501, L/W ratio 1.39; dorsal plate L 661; frontal plate L 142-147, W 50, L/W ratio 2.8-3.0; gnathosomal bay L 170, Cx-I total L 313, Cx-I medial L 142, Cx-II+III medial 52; ratio Cx-I L/Cx-II+III medial L 6.1; Cx-I medial L/Cx-II+III medial L 2.7; distance between glandular openings of Cxgl-4 and Cxgl-2 51–54. Genital field L/W 171/150, L/W ratio 1.14; distance genital field–excretory pore 181, genital field–caudal idiosoma margin 305. Gnathosoma vL 359; chelicera total L 434; palp total L 358, dL: P-1, 46; P-2, 114; P-3, 69; P-4, 109; P-5, 20; P-2/P-4 ratio 1.05.

##### Remarks.

*Torrenticola ussuriensis* was originally described by Sokolow (1934) from the Primory Territory in the Russian Far East, and later reported from River Inôzava in Japan (Enami 1940). Recently this species was redescribed by Pešić et al. (2011) based on new material from the Russian Far East. A single female specimen examined from River Inje agrees well with the redescription of *Torrenticola ussuriensis*. The only difference is found in the excretory pore lying on the same level as Vgl-2 in specimen from Korea while in specimens from Russia and Japan (see: Enami 1940) the excretory pore is shifted slightly posterior to Vgl-2.

##### Habitat.

A permanent sandy/bouldery river with considerably exposure to sunlight (Fig. 14E).

##### Distribution.

Far East of Russia (Primory and Khabarovsk Territory, Jewish Autonomous and Amurskaya Area - “*Atractides semisutus*” Sokolow 1934; Pešić et al. 2011); Japan (Uzi region – “*Atractides semisutus*” Enami 1940). New for the fauna of Korea.

#### Torrenticola turkestanica

(Sokolow, 1926)

http://species-id.net/wiki/Torrenticola_turkestanica

Figs 10 , 11G–H

Atractides turkestanicus Sokolow, 1926: 74.

##### Material examined.

SOUTH KOREA: CR3 River Inje, 38°03.961'N, 128°10.516'E, 225 m asl., 8.x.2012 Pešić & Karanović 1/1/0 (mounted, NIBRIV0000268852).

##### Morphology.

**Figure 10.**
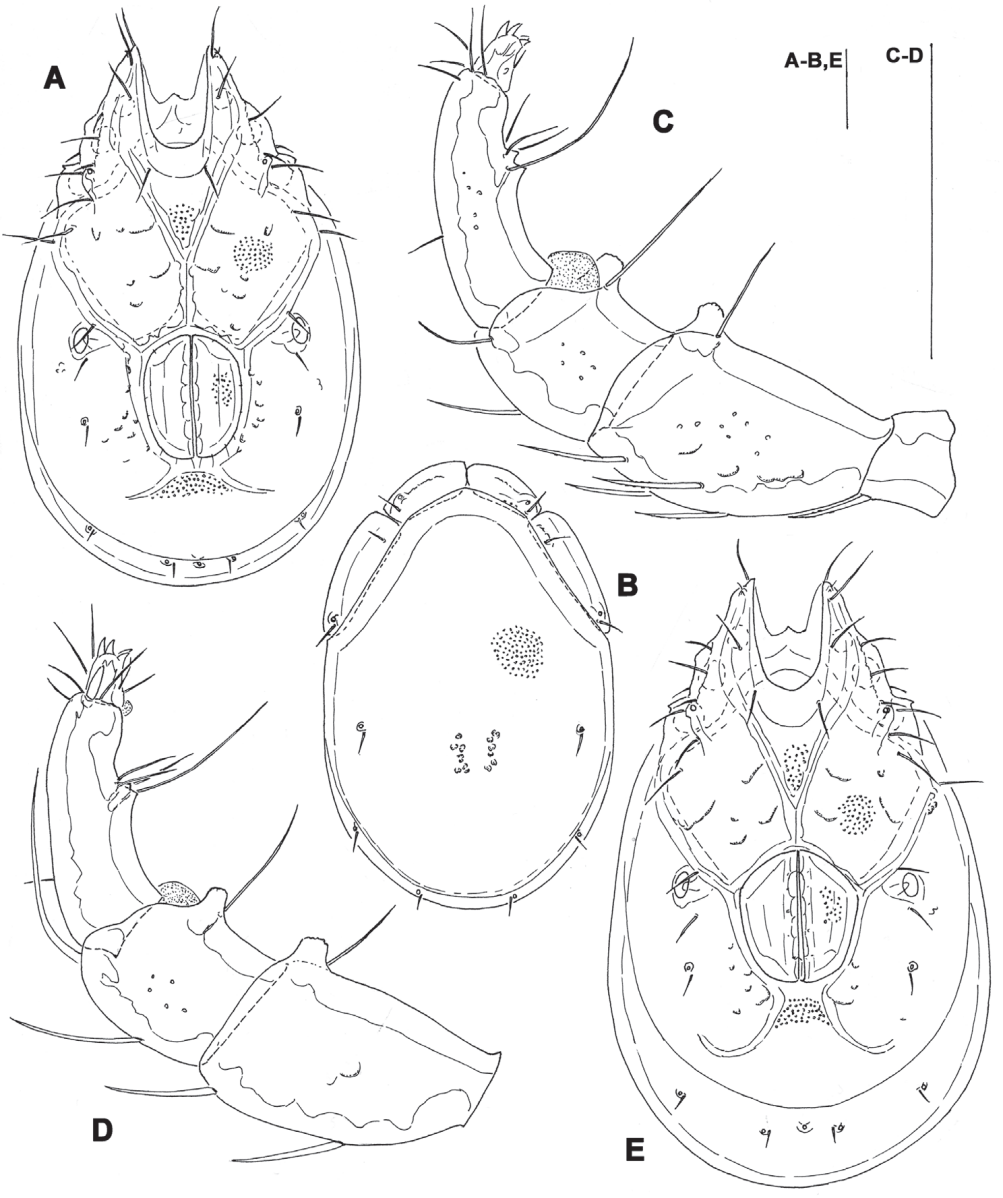
*Torrenticola turkestanica* (Sokolow, 1926) (**A–D** male **E** female), Inje River, Korea: **A, E** ventral shield **B** dorsal shield **C** palp, lateral view **D** palp, medial view (P-1 missing). Scale bars = 100 μm.

**Figure 11.**
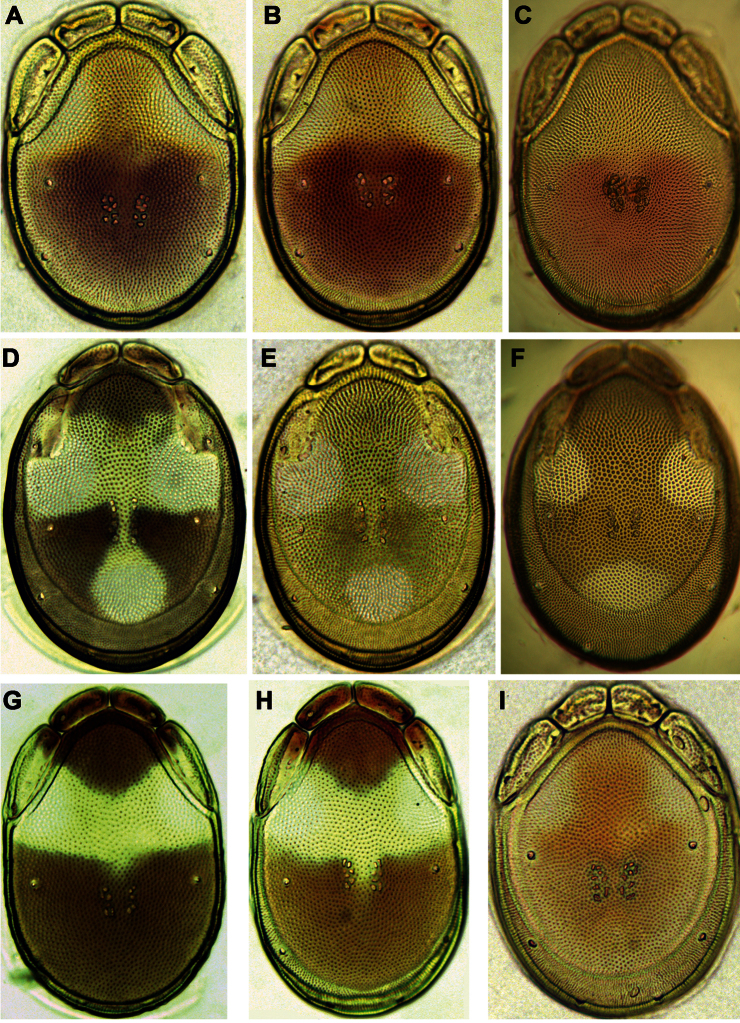
Photographs of dorsal shield: **A–C** *Torrenticola recentis* Tuzovskij, 2003 (**A, B** specimens from Dobong stream, Korea **C** specimen from River Kedrovaya, Russia): **A** male **C–B** female **D–F** *Torrenticola ussuriensis* (Sokolow, 1940), female (**D** specimen photographed immediately after dissection **E–F** specimens mounted in Hoyer’s medium): **D–E** specimen from Korea **F** specimen from Russia **G–H** *Torrenticola turkestanica* (Sokolow, 1926), specimens from River Inje, Korea: **G** male **H** female **I** *Monatarctides abei* sp. n., male holotype. Photos. V. Pešić (Figs **A–B, D–E, G–I**), K. Semenchenko (Figs **C, F**).

*General features*. Idiosoma elongated (dorsal shield L/W ratio 1.5-1.6); dorsal shield with colour pattern as illustrated in Figs 11G-H; Cxgl-4 subapical; gnathosoma ventral margin strongly curved; P-2 ventral margin convex, P-2 and P-3 with a subrectangular, apically serrated ventrodistal projection, P-4 stocky with well developed ventral protuberance bearing one long and three short setae (Figs 10C-D). Male: Medial suture line of Cx-II+III moderately long; genital field subrectangular in shape, ejaculatory complex normal in shape; excretory pore and Vgl-2 located on the margin of primary sclerotization. Female: Posterior suture line of Cx-IV curved and well evident; genital field excretory pore and Vgl-2 away from the line of primary sclerotization.

*Measurements*. Male: Idiosoma (ventral view: Fig. 10A) L 700, W 441; dorsal shield (Figs 7B, 11G) L 575, W 375, L/W ratio 1.5; dorsal plate L 541; shoulder plate L 172-174, W 48-56, L/W ratio 3.1-3.6; frontal plate L 106-116, W 44-47, L/W ratio 2.4-2.5; gnathosomal bay L 133, Cx-I total L 270, Cx-I medial L 137, Cx-II+III medial 97; ratio Cx-I L/Cx-II+III medial L 2.8; Cx-I medial L/Cx-II+III medial L 1.4. Genital field L/W 156/122, ratio 1.28; distance genital field–excretory pore 137, genital field–caudal idiosoma margin 169; ejaculatory complex L 212. Gnathosoma vL 283; chelicera total L 323; palp total L 272, dL: P-1, 28; P-2, 88; P-3, 52; P-4, 84; P-5, 20; P-2/P-4 ratio 1.05.

Female. Idiosoma (ventral view: Fig. 10E) L 781, W 494; dorsal shield (Fig. 11H) L 650, W 409, L/W ratio 1.6; dorsal plate L 610; shoulder plate L 178-189, W 56-64, L/W ratio 3.0-3.2; frontal plate L 127-131, W 52-55, L/W ratio 2.3-2.5; gnathosomal bay L 145, Cx-I total L 297, Cx-I medial L 151, Cx-II+III medial 47; ratio Cx-I L/Cx-II+III medial L 6.3; Cx-I medial L/Cx-II+III medial L 3.2. Genital field L/W 169/151, ratio 1.12; distance genital field–excretory pore 188, genital field–caudal idiosoma margin 272. Gnathosoma vL 372; chelicera total L 316; palp total L 301, dL: P-1, 31; P-2, 99; P-3, 59; P-4, 92; P-5, 20; P-2/P-4 ratio 1.08.

##### Remarks.

The specimens examined from River Inje fit the description of *Torrenticola turkestanica*, a species described based on a single female from Tadjikistan (Sokolow 1926). The only difference with the original description is found in a broader genital field in the type specimen. Later on, populations provisionally assigned to *Torrenticola turkestanica*, mainly based on the approved non-identity with the alternative species, were reported from Indian Himalayas (Pešić et al. 2007) and Thailand (Pešić and Smit 2006). The populations from Thailand differ from our material and the original description in a less slender idiosoma (dorsal shield L/W ratio 1.3-1.4, data taken from Pešić and Smit 2006), a much shorter ventral seta on P-2 and more slender P-4, and very likely represent an new species. The additional material and finding of a male from the locus typicus is necessary to clarify the taxonomy of *Torrenticola turkestanica* (the holotype may be missing, not found in the Zoological Institute of St. Petersburg, Denis Tumanov pers. comm).

*Torrenticola japonica* Imamura, 1953, a species described based on a single female from a stream in Shinjô-mura in Japan (Imamura 1953), resembles *Torrenticola turkestanica* in the characteristic shape of the palp (ventral margin of P-2 convex, P-2 and P-3 with subrectangular ventrodistal projection). From the latter species, *Torrenticola japonica* differs in a broader idiosoma, a dorsal shield with broader shoulder platelets and posterior suture line of Cx-IV not extending far beyond genital field (see: Imamura 1953). However as the holotype of *Torrenticola japonica* is probably lost (Hiroshi Kajihara, pers. comm), additional material and selection of a neotype from the locus typicus is necessary to clarify its taxonomy.

Imamura (1953) reported a single male of *Torrenticola elliptica* Maglio, 1909, from a stream in Shinjô-mura in Japan. However due to the presence of subrectangular ventrodistal projections on P-2 and P-3 and a convex ventral margin of P-2, his illustrations show a general conformity with *Torrenticola japonica* and *Torrenticola turkestanica*. It is very likely that the specimen attributed by Imamura (1953) to *Torrenticola elliptica* is conspecific with *Torrenticola japonica*, especially as both species were collected from the same location and on the same day.

##### Habitat.

A permanent sandy/bouldery river with considerable exposure to sunlight (Fig. 14E).

##### Distribution.

Tadjikistan (Sokolow 1926), Indian Himalayas (Pešić et al. 2007), Thailand (Pešić and Smit 2006, but see remarks above). New for the fauna of Korea.

### Genus *Monatractides* K. Viets, 1926

#### Monatractides abei sp. n.

urn:lsid:zoobank.org:act:F9C4384E-4673-4FC9-B098-A002FCDD1CDA

http://species-id.net/wiki/Monatractides_abei

Figs 12 , 13 , 11I

##### Type series.

Holotype male (NIBRIV0000268853), dissected and slide mounted, SOUTH KOREA, CR2 Seoul, Ui-dong stream, 37°39.554'N, 127°00.249'E, 114 m asl., 7.x.2012, Pešić & Choi. Paratypes: SOUTH KOREA, CR1 Seoul, Dobong stream, 37°41.262'N, 127°01.706'E, 19 m asl., 7.x.2012, Pešić & Choi, one female (NIBRIV0000268854), dissected and slide mounted; RUSSIA, Primory Territory, Partizansky District, Partizanskay River basin, Tigrovaya River, 43°11.401'N, 133°12.660'E; depth 30 cm below the sediment surface; substrate: cobbles, pebbles, sand; 12.vi.2010, Semenchenko & Sidorov, one female (497-kas–IBSS), dissected and slide mounted.

##### Diagnosis.

Lateral margins of dorsal shield nowhere subparallel; distal margins of P-3 with several pointed extensions; genital field in male with slightly protruding anteriolateral angles; medial suture line of Cx-II+III in female relatively long (L 90–105 μm).

##### Description.

**Figure 12.**
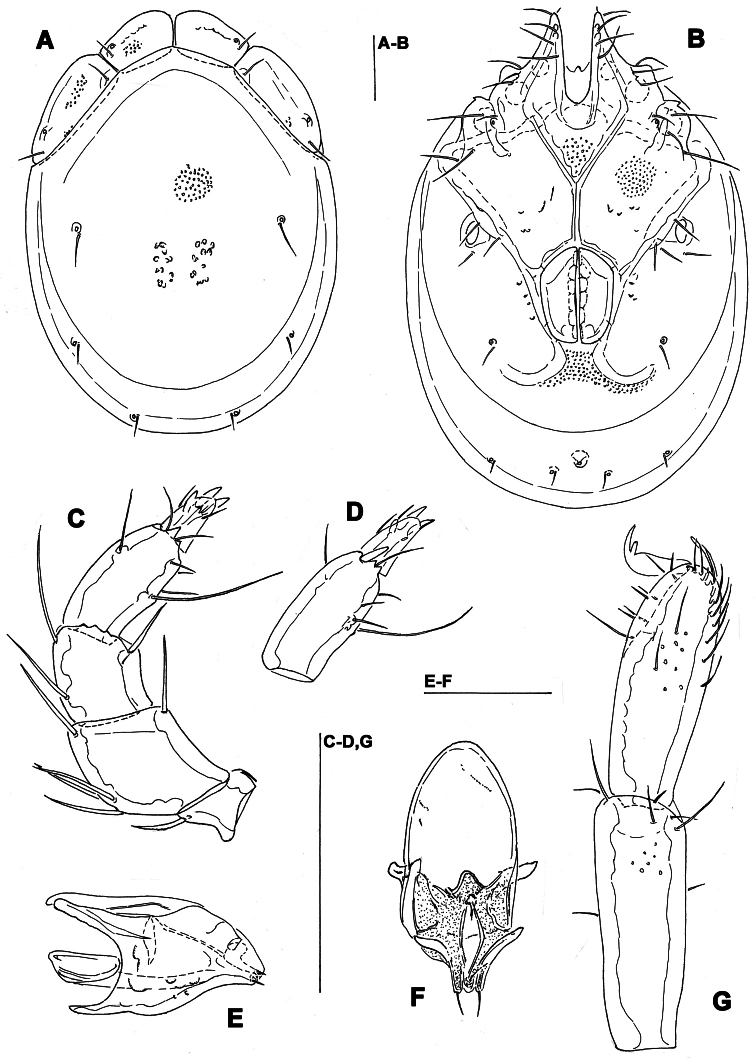
*Monatractides abei* sp. n., male holotype: **A** dorsal shield **B** ventral shield **C** palp, lateral view **D** P-4 and -5, medial view **E** gnathosoma and chelicera **F** ejaculatory complex **G** I–Leg-5 and -6. Scale bars = 100 μm.

*General features*. Lateral margins of dorsal shield nowhere subparallel (Fig. 12A, 11I); three pairs of knob-like protrusions on the lateral margin of gnathosomal bay; suture line of Cx-IV distinct, originating from lateral edge of genital field, laterally curved anteriorly; excretory pore away from the line of primary sclerotization, Vgl-2 posterior to excretory pore; ejaculatory complex (Fig. 12F): proximal chamber large, proximal horns reduced; P-2 equal in length, or only slightly shorter than P-4; distal margins of P-3 with several pointed extensions; ventral seta on P-4 relatively long and away from distal edge (Figs 12C-D). Male. Medial suture line of Cx-II+III moderately long, genital field with slightly protruding anteriolateral angles. Female: Similar to the male; the short postgenital area and caudal position of the excretory pore in the specimen from Korea are due to the obviously juvenile age (indicated by weak sclerotization and absence of eggs); medial suture line of Cx-II+III relatively long.

*Measurements*. Male: Idiosoma (ventral view: Fig. 12B) L 775, W 538; dorsal shield (Fig. 12A, 11I) L 663, W 488, ratio 1.36; dorsal plate L 606; shoulder plate L 191-194, W 75, ratio 2.5-2.6; frontal plate L 122-123, W 75, ratio 1.6; shoulder/frontal plate L ratio 1.6. Gnathosomal bay L 143, Cx-I total L 264, Cx-I mL 120, Cx-II+III mL 99; ratio Cx-I L/Cx-II+III mL 2.7; Cx-I mL/Cx-II+III mL 1.2. Genital field L/W 147/115, ratio 1.28; ejaculatory complex L 197; distance genital field-excretory pore 191, genital field-caudal idiosoma margin 264. Gnathosoma vL 152; chelicera total L 181; palp total L 199, dL: P-1, 23; P-2, 54; P-3, 40; P-4, 54; P-5, 28; P-2/P-4 ratio 1.0; dL of I-Leg-5-6 (Fig. 9G): 99, 97.

**Figure 13.**
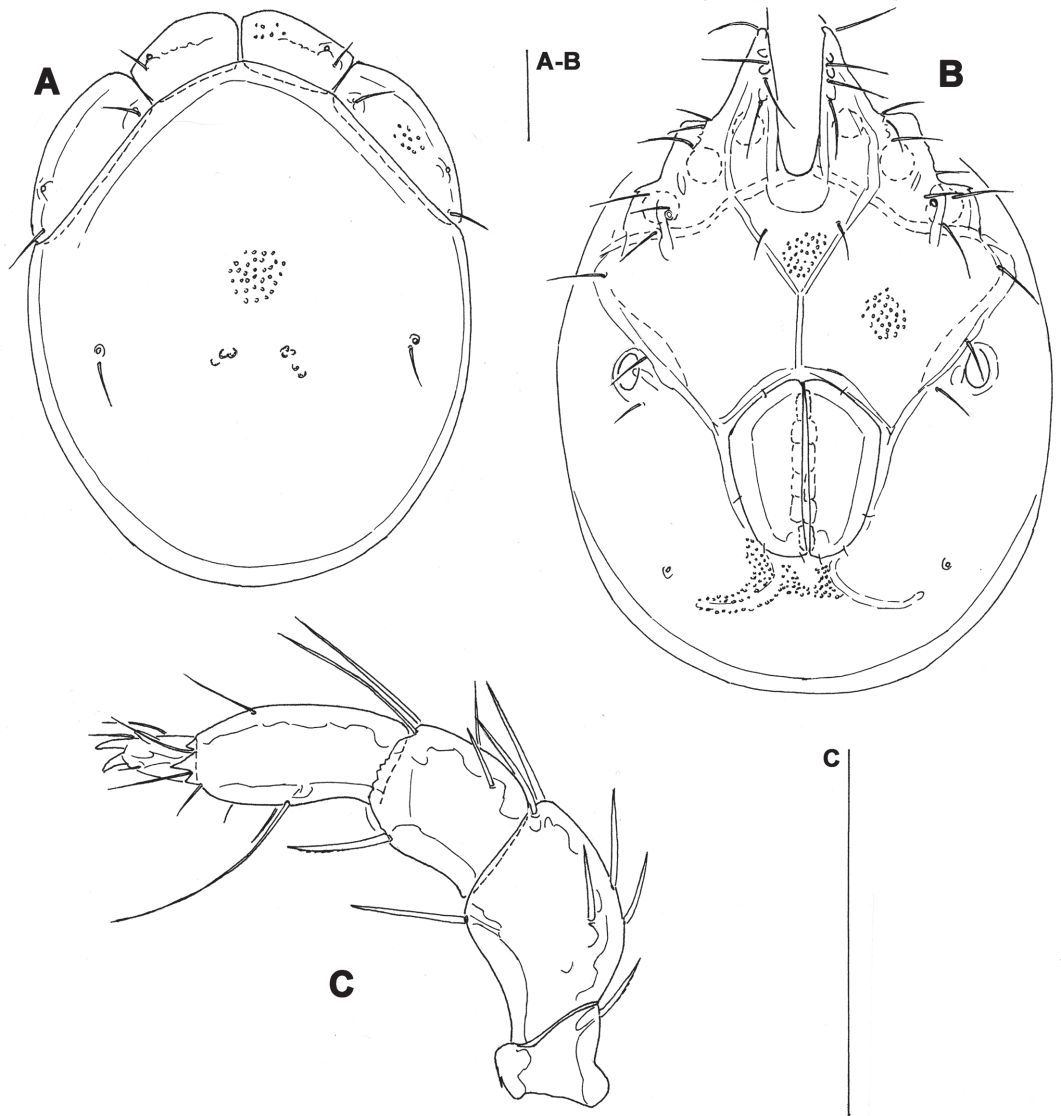
*Monatractides abei* sp. n., paratype female, Dobong stream, Korea: **A** dorsal shield **B** ventral shield **C** palp, lateral view. Scale bars = 100 μm.

Female (from CR2, in parentheses specimen from Russia). Idiosoma (ventral view: Fig. 13B) L 725 (800), W 544 (612); dorsal shield (Fig. 13A) L 625 (680), W 488 (476), L/W ratio 1.28 (1.43); dorsal plate L 575 (620); shoulder plate L 203-206 (204), W 70-76 (74), L/W ratio 2.7-2.9 (2.76); frontal plate L 125-127 (114), W 69-70 (79), ratio 1.8 (1.43); shoulder/frontal plate L ratio 1.6 (1.79). Gnathosomal bay L 159 (132), Cx-I total L 283 (257), Cx-I mL 123 (118), Cx-II+III mL 90 (105); ratio Cx-I L/Cx-II+III mL 3.1 (2.45); Cx-I mL/Cx-II+III mL 1.4 (1.1). Genital field L/W 191 (145) /160 (112), ratio 1.19 (1.28); distance genital field-excretory pore (205), genital field-caudal idiosoma margin (277). Gnathosoma vL 162 (198); chelicera total L 202 (200); palp total L 209, dL: P-1, 28; P-2, 56 (54); P-3, 39 (40); P-4, 58 (54); P-5, 28 (19); P-2/P-4 ratio 0.97 (1.0); dL of I-Leg-4-6: 114 (103), 105 (100), 104 (94).

**Figure 14.**
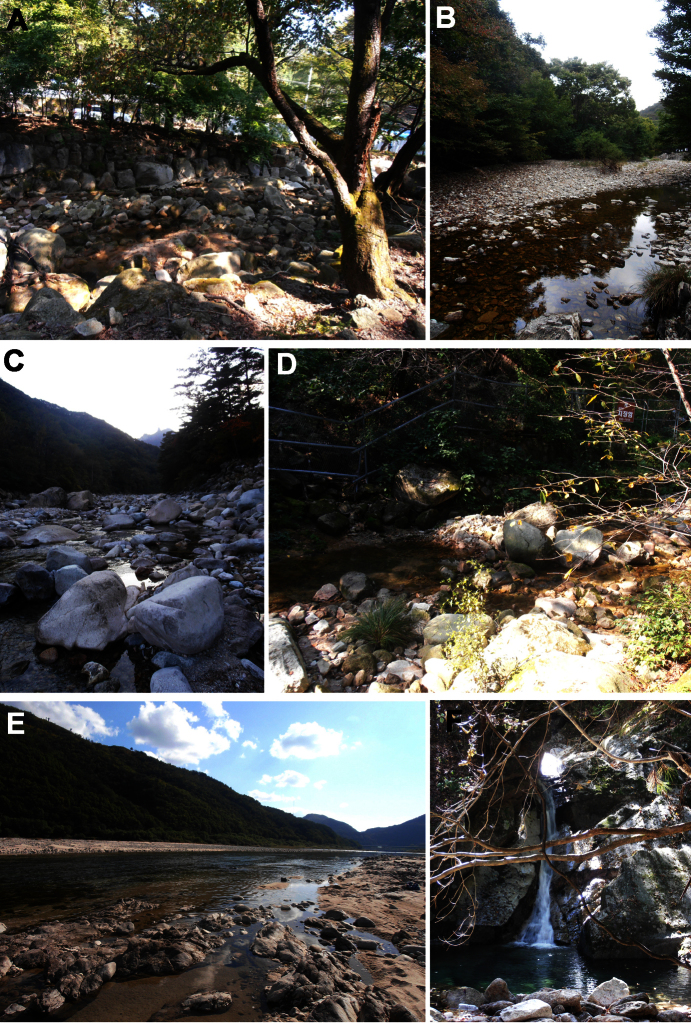
Photographs of selected sampling sites. **A** CR1 (Dobong stream, sampling site of *Torrenticola recentis*, *Torrenticola nipponica* and *Monatractides abei* sp. n.) **B** CR9 (stream in Naebyeansan NP, sampling site of *Torrenticola brevirostris* and *Torrenticola dentifera*);] **C** CR4 (stream in Seoraksan NP, type locality of *Torrenticola kimichungi* sp. n. and sampling site of *Torrenticola recentis*) **D** CR2 (Ui-dong stream, type locality of *Monatractides abei* sp. n. and sampling site of *Torrenticola recentis*) **E** Inje River (sampling site of *Torrenticola ussuriensisis* and *Torrenticola turkestanica*) **F** CR12 (stream in JiriSan NP, sampling site of *Torrenticola recentis*). Photos. V. Pešić.

##### Etymology.

The species is named after Dr Hiroshi Abe in appreciation of his studies on water mites.

##### Remarks.

*Monatractides abei* sp. n. is apparently closely related to *Monatractides madritensis* (K. Viets, 1930), known from the Western Palaearctic, due to the presence of an elongated ventral seta on P-4 and the similar shape of the ejaculatory complex. Males of *Monatractides madritensis* differ in a more slender idiosoma with subparallel lateral margins of the dorsal shield, and a slender genital field with more protruding anterior margins of the genital flaps forming a more acute angle (see: Di Sabatino et al. 2010). Furthermore, in *Monatractides abei* nov. sp. the distal margins of P-3 bears several pointed extensions. The female of the new species can be identified on the basis of a relatively long medial suture line of Cx-II+III.

Enami (1940) stated that he identified *Monatractides* population from River Inôzava (Uzi region, Japan) as *Torrenticola stadleri* (syn. to *Monatractides stadleri*), but he noted that his specimens differ from the later species in P-4 bearing an elongated ventral seta. Given the latter character and the shape of genital field in Enami’s specimens, with slightly protruding anteriolateral angles in male, resembling the female, suggest that these specimens are probably conspecific with *Monatractides abei* sp. n.

##### Habitat.

The specimens of *Monatractides abei* sp. n. was collected in two sandy/bouldary streams, shaded by riparian vegetation (Fig. 14A, D); the specimens from Russia were collected from interstitial waters.

##### Distribution.

South Korea, Far East of Russia (present study).

## Supplementary Material

XML Treatment for Torrenticola brevirostris

XML Treatment for Torrenticola dentifera

XML Treatment for Torrenticola kimichungi

XML Treatment for Torrenticola nipponica

XML Treatment for Torrenticola recentis

XML Treatment for Torrenticola ussuriensis

XML Treatment for Torrenticola turkestanica

XML Treatment for Monatractides abei
